# Identifying novel therapeutic targets for non-alcoholic fatty liver disease using bioinformatics approaches: from drug repositioning to traditional Chinese medicine

**DOI:** 10.3389/fbinf.2025.1613985

**Published:** 2025-08-26

**Authors:** Jingmin Zhang, Tianwei Meng, Weiqi Gao, Xinghua Li, Juan Xu

**Affiliations:** ^1^ Shanxi Bethune Hospital, Shanxi Academy of Medical Sciences, Third Hospital of Shanxi Medical University, Tongji Shanxi Hospital, Taiyuan, China; ^2^ Heilongjiang University of Chinese Medicine, Harbin, Heilongjiang, China; ^3^ Department of Pharmacy, Changzhi People’s Hospital, Changzhi, China

**Keywords:** non-alcoholic fatty liver disease, bioinformatics, drug repositioning, traditional Chinese medicine, cyclosporine

## Abstract

**Background:**

Non-alcoholic fatty liver disease (NAFLD) is a prevalent condition with limited effective treatments, necessitating novel therapeutic strategies. Bioinformatics offers a promising approach to identify new targets by analyzing gene expression and drug interactions.

**Objective:**

This study aims to identify novel therapeutic targets for NAFLD through bioinformatics, focusing on drug repositioning and traditional Chinese medicine (TCM) components.

**Methods:**

Three NAFLD-related gene expression datasets (GSE260666, GSE126848, GSE135251) were analyzed to identify differentially expressed genes. Protein-protein interaction networks were constructed using STRING and visualized with Cytoscape. Pathway enrichment analysis was performed, and drug-gene interactions were explored using the DGIdb database. TCM components were screened via the HERB database, with molecular docking conducted to assess binding affinities.

**Results:**

Key hub genes (CXCL2, CDKN1A, TNFRSF12A, HGFAC) were identified, with significant enrichment in cell proliferation and PI3K-Akt signaling pathways. Cyclosporine emerged as a potential repurposed drug, while TCM components (curcumin, resveratrol, berberine) showed strong binding affinities to NAFLD targets.

**Conclusion:**

Cyclosporine and TCM compounds are promising candidates for NAFLD treatment, warranting further experimental validation to confirm their therapeutic potential.

## 1 Introduction

Nonalcoholic fatty liver disease (NAFLD) is a multifactorial and complex disorder recognized as a significant etiological factor for liver cancer (McVey et al., 2022), although its pathogenesis remains incompletely understood (Wang et al., 2017). NAFLD is prevalent globally, with an estimated prevalence of approximately 30% in the general population and a significantly higher prevalence in males (40% vs. 26%) (Zhang et al., 2021; Riazi et al., 2022; Younossi et al., 2023). Most patients with NAFLD remain asymptomatic; however, individuals progressing to metabolic-associated steatohepatitis may experience symptoms such as fatigue, malaise, and vague discomfort in the right upper quadrant (Younossi et al., 2011; Le et al., 2023). The long-term prognosis of NAFLD is primarily influenced by the presence of atherosclerotic cardiovascular disease and extrahepatic malignancies. In cases of advanced fibrosis, liver-related events, including hepatic decompensation, hepatocellular carcinoma, liver transplantation, and liver-related mortality, increase significantly (Sun et al., 2023; Toader et al., 2025). Current therapeutic strategies for NAFLD primarily encompass lifestyle modifications and pharmacological interventions, including weight reduction, antihypertensive agents, and hypoglycemic medications (Tsamos et al., 2023). However, due to limited efficacy and generally suboptimal clinical outcomes, the development of novel therapeutic agents remains a critical area of research.

Drug repositioning entails identifying new indications for approved drugs (Wang et al., 2025). This approach is distinguished by its low risk and high efficiency, effectively addressing the challenges of high costs and low success rates in new drug development while expediting the market availability of therapeutic agents, particularly for rare diseases (Hurle et al., 2013). Drug repositioning has evolved from traditional random screening for new indications to a more precise, computer-assisted research phase. Nevertheless, computational drug-repositioning efforts are hindered by algorithmic bias arising from incomplete or noisy omics data, heterogeneous disease definitions, and the frequent absence of prospective experimental validation, all of which can limit the translational success of *in silico* predictions (Loscalzo, 2024).This process involves integrating data from small-molecule ligand and protein receptor databases, along with experimental validation, to evaluate the feasibility of repurposing existing drugs for new indications.

The Drug-Gene Interaction Database (DGIdb) is a specialized and efficient resource that integrates drug-gene interaction relationships (Huang et al., 2024). DGIdb can be accessed through a programmatic interface or its web-based user interface for interactive operations. The HERB database (Fang et al., 2021) analyzes the gene expression profiles of traditional Chinese medicines (TCMs) and their components, correlating these profiles with the world’s largest pharmacogenomics database, the Connectivity Map, to establish systematic links between TCM components and modern drugs. In this study, bioinformatics methods were employed to investigate differentially expressed genes associated with NAFLD, thereby identifying novel therapeutic agents for NAFLD and providing new directions for subsequent drug development.

## 2 Materials and methods

### 2.1 Acquisition of gene expression microarray data and screening for differentially expressed genes

Relevant microarray datasets associated with NAFLD were retrieved from the GEO microarray database (Clough et al., 2024) (GEO, https://www.ncbi.nlm.nih.gov/geo/) using the keywords “non-alcoholic fatty liver disease” and “NAFLD”. After comparison and screening, three datasets (GSE260666, GSE126848, and GSE135251) were ultimately selected for the study. Only human-liver RNA-seq studies providing raw counts, ≥10 samples and full NAFLD histology without drug-treatment confounders met the eligibility criteria. To minimise cross-study batch effects, differential expression was calculated inside each dataset with GEO2R; subsequent analyses used the intersection of significant genes across the three datasets, a strategy that bypasses direct data merging and reduces technical bias. Expression data from NAFLD liver tissues and normal liver tissues were extracted from the samples for comparative analysis. Differential expression was determined using a combined criterion of |log_2_FC| ≥ 1 and an adjusted *P* value <0.05; genes meeting these thresholds were classified as significantly up- or downregulated accordingly. Finally, the intersection of differentially expressed genes from the three datasets was identified, and the shared genes were designated as the key differentially expressed genes (DEGs) for this study.

### 2.2 Construction and visualization of protein-protein interaction networks

The differentially expressed genes were entered into the STRING database (Franceschini et al., 2013) (https://string-db.org/) to construct a protein-protein interaction (PPI) network, with an interaction threshold set at 0.400, which corresponds to STRING’s medium-confidence score and is widely used to achieve a balance between capturing biologically meaningful interactions and minimizing false positives in large-scale analyses, to identify proteins with significant interactions. Subsequently, the PPI network was visualized using Cytoscape software (version 3.9.1) (Shannon et al., 2003), and highly interconnected protein interaction modules were identified using the MCODE plugin to create corresponding visual network diagrams. The specific parameters were set as follows: degree = 2, node score cutoff = 0.2, k-core = 2, and maximum depth = 100.

### 2.3 GO enrichment analysis and KEGG pathway analysis of differentially expressed genes

The Omicshare online analysis tool (http://www.omicshare.com/tools/index.php/) was utilized to perform Gene Ontology (GO) secondary classification enrichment analysis and Kyoto Encyclopedia of Genes and Genomes (KEGG) pathway enrichment analysis on differentially expressed genes. Enrichment significance was assessed with the hypergeometric test, and the resulting *P* values were corrected for multiple comparisons using the Benjamini–Hochberg false-discovery rate method; pathways with an adjusted *P* < 0.05 were considered significant. The species selected for this analysis was *Homo sapiens*, and the results included biological processes, molecular functions, and cellular components from the GO enrichment analysis, as well as related biological pathway enrichment outcomes.

### 2.4 Analysis of drug-gene interactions

The identified differentially expressed genes associated with NAFLD were entered into the DGIdb database (https://dgidb.org) (Cannon et al., 2024) to identify potential drugs that may target these core genes. Drugs predicted to exhibit known interaction types with the core genes of NAFLD are considered potential candidates for treating this condition. Subsequently, the interactions between the potential drugs and their corresponding target genes were visualized using Cytoscape software.

### 2.5 Screening and prediction of potential traditional Chinese medicine components for the treatment of NAFLD

The differentially expressed genes were entered into the HERB database (Fang et al., 2021) (http://herb.ac.cn/) to filter out traditional Chinese medicine components that have a statistically significant mapping relationship (*P* < 0.05) with these genes. A statistical analysis was conducted on these traditional Chinese medicine components to examine the distribution of their meridian affinities and efficacy categories.

### 2.6 Molecular docking

The three-dimensional (3D) structure of the target compound was downloaded from the PubChem database (https://pubchem.ncbi.nlm.nih.gov/). Target protein structures were then selected from the Protein Data Bank based on the following criteria: a resolution below 2.5 Å and the presence of co-crystallized small-molecule ligands. Subsequently, the selected compounds and target proteins were submitted to the receptor-ligand docking platform DockThor (https://dockthor.lncc.br/v2/) for molecular docking analysis. The top-ranked docking results were visualized using PyMOL 2.4.0 software. The binding stability between the molecules was assessed by analyzing the docking affinity scores.

## 3 Results

### 3.1 Selection and screening of gene expression data

The gene expression data selected for this study includes: ① A study published in 2004 by Xiangya Second Hospital of Central South University, which analyzed the differential gene expression in liver tissues of patients with NAFLD compared to a control group. The dataset is identified as GSE260666 (https://www.ncbi.nlm.nih.gov/geo/query/acc.cgi?acc=GSE260666), comprising a total of 16 samples, including 10 NAFLD samples and 6 normal liver tissue samples; ② A transcriptomic analysis across the spectrum of non-alcoholic fatty liver disease published in 2020 by Newcastle University, with the dataset identified as GSE135251 (https://www.ncbi.nlm.nih.gov/geo/query/acc.cgi?acc=GSE135251), comprising a total of 216 samples, including 206 samples at various stages of fibrosis and 10 normal liver tissue samples; ③ A study published in 2019 by Gubler in Denmark that compared the hepatic transcriptomic characteristics of patients with varying degrees of non-alcoholic fatty liver disease to those of healthy individuals with normal weight. The dataset is identified as GSE126848 (https://www.ncbi.nlm.nih.gov/geo/query/acc.cgi?acc=GSE126848), comprising a total of 57 samples, including 14 normal healthy liver tissue samples, 12 normal healthy obese liver tissue samples, 15 samples of simple steatosis, and 16 samples of non-alcoholic steatohepatitis with or without fibrosis.

### 3.2 Acquisition and selection method of differentially expressed genes

**FIGURE 1:**
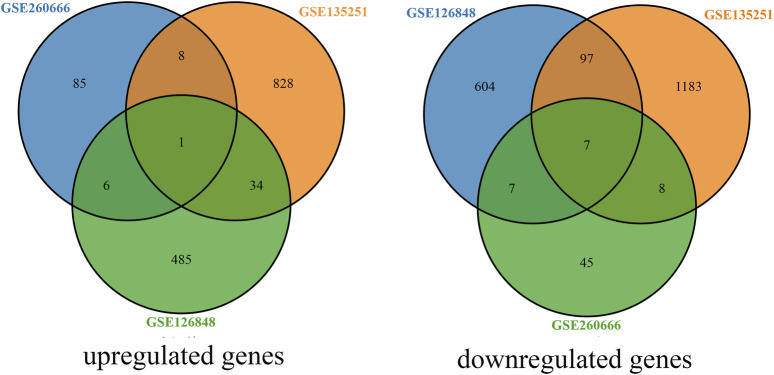
Venn diagram of differentially expressed genes.

Using *P* < 0.05 and |log2FC| ≥ 1 as the criteria for selection, a total of 167 genes were identified in the dataset GSE260666, including 100 upregulated genes and 67 downregulated genes. In the dataset GSE135251, a total of 2,166 genes were identified, including 871 upregulated genes and 1,295 downregulated genes. In the dataset GSE126848, a total of 1,241 genes were identified, including 526 upregulated genes and 715 downregulated genes. By constructing a Venn diagram to analyze the intersection of upregulated and downregulated genes from the three datasets, a total of 34 differentially expressed genes were identified, including 16 upregulated and 18 downregulated genes (Figure 1).

### 3.3 Modular analysis of the protein-protein interaction network

**FIGURE 2:**
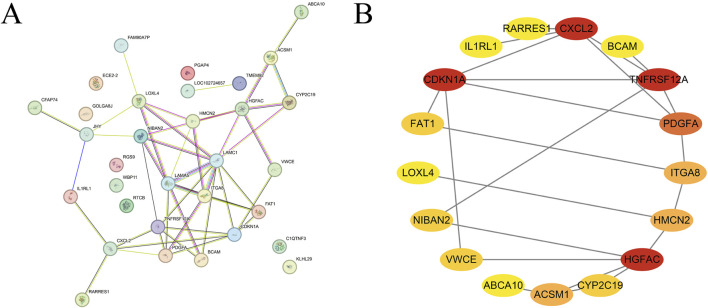
Protein-protein interaction network **(A)**. Module analysis and screening of core differentially expressed genes **(B)**.

A total of 34 differentially expressed genes were entered into the STRING database to construct the PPI network (Figure 2A), resulting in 22 interactions. The network was then visualized and analyzed for modularity using Cytoscape software (version 3.9.1) along with the MCODE plugin, identifying the most significant module, which is highlighted in red (Figure 2B). Further analysis revealed that CXCL2, CDKN1A, TNFRSF12A, and HGFAC are the key regulatory hub genes within this module of differentially expressed genes.

### 3.4 Enrichment analysis

**FIGURE 3:**
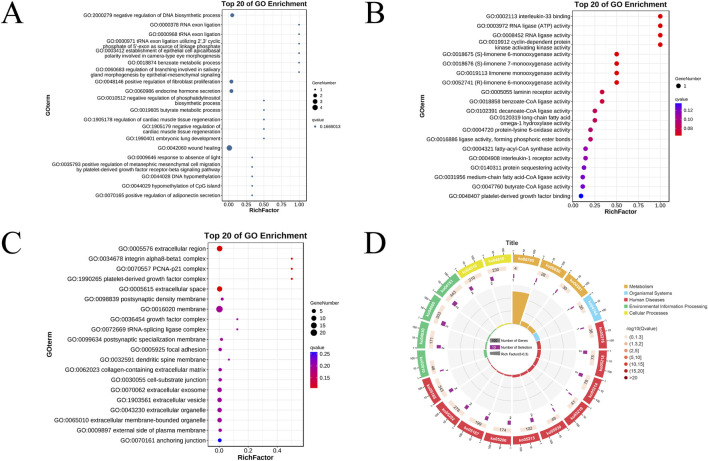
Bubble diagram of GO biological process enrichment analysis **(A)**. Bubble diagram of functional enrichment analysis of GO molecules **(B)**. Bubble chart of GO Cellular Component enrichment analysis **(C)**. The KEGG pathway enrichment circle plot places a scale for gene counts on its periphery; the first ring presents the enriched categories and pathway IDs, with red indicating human diseases, blue denoting biological systems, and green representing environmental information processing; in the second ring, bar length corresponds to the number of background genes and colour depth maps the P-value, darker shades signifying smaller values; the third ring displays the total number of foreground genes; the fourth ring charts the enrichment metric Rich Factor, where each minor gridline equals 0.1 and larger values denote stronger enrichment **(D)**.

This study employed a significance threshold of *P* < 0.05 for selection, resulting in a total of 231 entries from the GO enrichment analysis. Among these, 161 entries pertain to biological processes, including the negative regulation of DNA biosynthesis, RNA splicing, and modulation of fibroblast proliferation. Forty-four entries relate to molecular functions, encompassing interleukin-33 binding, interleukin-1 receptor activity, and cyclin-dependent protein kinase activating kinase activity. Additionally, 26 entries correspond to cellular components, which include integrin α8-β1 complexes, growth factor complexes, and the extracellular environment. The top 20 results for biological processes, molecular functions, and cellular components are illustrated in bubble charts (Figures 3A–C).

The KEGG enrichment analysis identified a total of 81 pathways, of which 17 exhibited a significance level of *P* < 0.05, as detailed in Table 1. The results indicate that these pathways are enriched in several tumor-related processes, including cytokine interactions, the PI3K-Akt signaling pathway, and butyrate metabolism, with the melanoma pathway showing the strongest correlation. The results of the KEGG enrichment analysis are presented in a circular diagram (Figure 3D).

**TABLE 1:** KEGG pathway enrichment analysis results.

Pathway	*P*-value
Melanoma	3.40 × 10^−3^
Glioma	3.77 × 10^−3^
Cytokine-cytokine receptor interaction	4.66 × 10^−3^
Lipoic acid metabolism	4.77 × 10^−3^
Prostate cancer	6.21 × 10^−3^
PI3K-Akt signaling pathway	1.34 × 10^−2^
JAK-STAT signaling pathway	1.68 × 10^−2^
MicroRNAs in cancer	1.73 × 10^−2^
Kaposi sarcoma-associated herpesvirus infection	2.23 × 10^−2^
Focal adhesion	2.47 × 10^−2^
Regulation of actin cytoskeleton	2.92 × 10^−2^
Butanoate metabolism	3.30 × 10^−2^
Linoleic acid metabolism	3.53 × 10^−2^
Phototransduction	3.53 × 10^−2^
Transcriptional misregulation in cancer	4.15 × 10^−2^
Thyroid cancer	4.22 × 10^−2^
Bladder cancer	4.79 × 10^−2^

### 3.5 Analysis of drug-gene interactions

**FIGURE 4:**
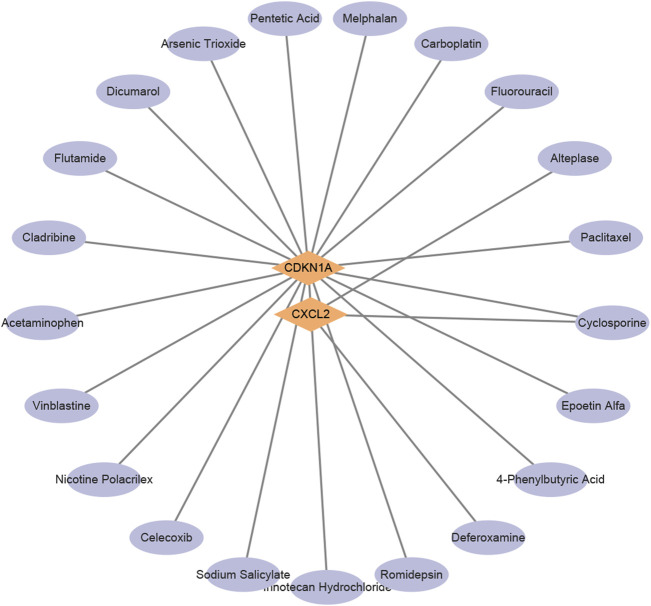
Predicted potential drug interactions with core genes.

To explore potential therapeutic strategies, this study utilized the DGIdb database to predict drugs that target the aforementioned core genes (Figure 4). The selection criteria included drugs approved by the U.S. Food and Drug Administration, resulting in a list of chemical compounds (Table 2). A total of 22 drugs or compounds targeting the core genes CDKN1A and CXCL2 were identified. However, no drugs were predicted to potentially regulate the HGFAC gene. The U.S. Food and Drug Administration has not yet approved any drugs or compounds targeting TNFRSF12A. To validate its causal role in NAFLD pathogenesis, an initial step could involve pooled CRISPR-Cas9 knockout screens in human hepatocyte-like cells subjected to lipotoxic stress. Subsequent validation would include arrayed siRNA knock-down assays in hepatic organoids derived from NAFLD patients. Key convergent phenotypic readouts such as neutral lipid accumulation, NF-κB signaling activity, and pyroptotic cytokine release could then be assessed to confirm mechanistic involvement and identify potential assay formats suitable for future small-molecule discovery. Additionally, cyclosporine shows potential activity against CDKN1A and CXCL2, making it a particularly promising novel candidate compound for NAFLD treatment.

**TABLE 2:** Predicted drug–gene interactions and their DGIdb interaction scores.

Gene	Drug	Interaction score
CDKN1A	ARSENIC TRIOXIDE	0.13
CDKN1A	CLADRIBINE	0.22
CDKN1A	NICOTINE POLACRILEX	0.07
CDKN1A	IRINOTECAN HYDROCHLORIDE	0.05
CDKN1A	4-PHENYLBUTYRIC ACID	0.17
CDKN1A	CYCLOSPORINE	0.04
CDKN1A	FLUOROURACIL	0.04
CDKN1A	MELPHALAN	0.17
CDKN1A	DICUMAROL	0.42
CDKN1A	ACETAMINOPHEN	0.06
CDKN1A	CELECOXIB	0.05
CDKN1A	ROMIDEPSIN	0.12
CDKN1A	EPOETIN ALFA	0.10
CDKN1A	PACLITAXEL	0.03
CDKN1A	CARBOPLATIN	0.04
CDKN1A	PENTETIC ACID	1.12
CDKN1A	FLUTAMIDE	0.17
CDKN1A	VINBLASTINE	0.10
CDKN1A	SODIUM SALICYLATE	0.84
CXCL2	DEFEROXAMINE	0.51
CXCL2	CYCLOSPORINE	0.10
CXCL2	ALTEPLASE	0.41

### 3.6 Results of herbal component screening

After inputting the core genes CXCL2 and CDKN1A, the search results from the HERB database identified five components: curcumin, resveratrol, ursolic acid, berberine, and tetrandrine. Screening through the TCMSP database (https://www.tcmsp-e.com) revealed that these components can be derived from various TCMs, including *Alpinia officinarum* (Gaoliangjiang), *Curcuma longa* (Jianghuang), Polygonum cuspidatum (Huzhang), Smilax glabra (Tufuling), *Coptis chinensis* (Huanglian), *Stephania tetrandra* (Fangji), and *Cornus officinalis* (Shanzhuyu).

### 3.7 Molecular docking

**FIGURE 5:**
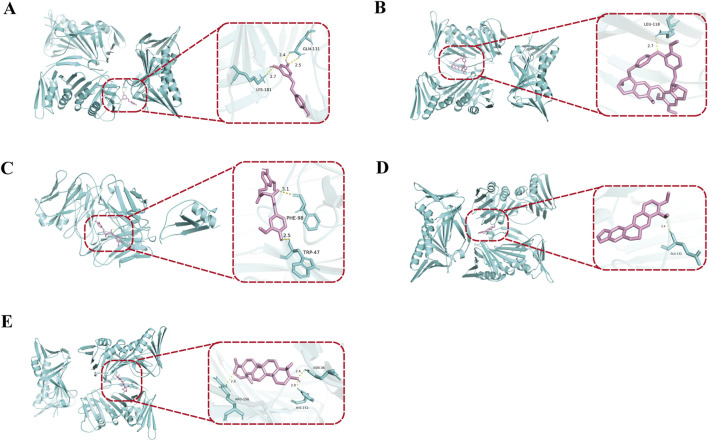
Molecular docking analysis of representative active compounds with core NAFLD-related targets. Curcumin was docked with CXCL2, forming hydrogen bonds with lysine at position 181 (2.7 Å), glutamine at position 131 (2.5 Å), and glutamic acid at position 131 (2.4 Å) **(A)**. Resveratrol interacted with CDKN1A, establishing a hydrogen bond with leucine at position 118 (2.7 Å) **(B)**. Ursolic acid bound to CDKN1A, forming hydrogen bonds with tryptophan at position 47 (2.5 Å) and phenylalanine at position 98 (3.1 Å) **(C)**. Berberine was docked with CDKN1A, generating a hydrogen bond with glutamic acid at position 112 (2.4 Å) **(D)**. Tetrandrine interacted with CDKN1A, forming hydrogen bonds with asparagine at position 36 (2.4 Å), histidine at position 152 (2.9 Å), and aspartic acid at position 196 (2.8 Å) **(E)**. These interactions demonstrate the strong binding affinities and structural stability between natural compounds and NAFLD-related targets.

This study employs molecular docking techniques to investigate the binding modes and characteristics of active components from traditional Chinese medicine with the core genes CXCL2 and CDKN1A. The three-dimensional structures of the proteins corresponding to the core genes were obtained from the Protein Data Bank database. As indicated in Table 3, the binding energies of all active components from traditional Chinese medicine with the target proteins are below −5 kcal/mol, suggesting a strong binding affinity between these components and the core targets (Wong et al., 2022). The visualization results of the molecular docking (Figures 5A–E) further reveal that these small molecules from traditional Chinese medicine can interact with key amino acid residues of the target proteins through hydrogen bonds, resulting in stable complex conformations. The findings of this study not only validate the potential mechanisms of action of these active components from traditional Chinese medicine at the molecular level concerning targets related to NAFLD but also provide a theoretical basis for their application in NAFLD treatment. Guided by the principles of traditional Chinese medicine, the aforementioned active components can be incorporated into modified prescriptions to support the precise treatment of NAFLD, thereby enhancing clinical efficacy.

**TABLE 3:** Binding energy of Ingredients to genes.

Ingredient name	Gene	Binding energy (kcal/mol)
curcumin	CXCL2	−7.5
resveratrol	CDKN1A	−7.1
ursolic acid	CDKN1A	−8.3
berberine	CDKN1A	−7.8
tetrandrine	CDKN1A	−8.8

## 4 Discussion

### 4.1 Impact of core differentially expressed genes on the liver

This study utilized gene expression data from the GEO database to identify differentially expressed genes associated with NAFLD. The analysis revealed a total of 16 upregulated and 18 downregulated genes, with CXCL2, CDKN1A, TNFRSF12A, and HGFAC exhibiting the highest composite scores.

CXCL2, also known as C-X-C motif chemokine ligand 2, is a chemokine that plays a crucial role in hepatic inflammatory responses. Studies indicate that the expression level of CXCL2 is significantly elevated in the liver tissues of NAFLD patients, primarily synthesized by intrahepatic macrophages (Han et al., 2022). Ke-Qi Han et al. (Han et al., 2015) found that inhibiting the expression of CXCL1 can slow the growth of liver tumors in nude mice and suppress the expression of CXCL2, CXCL3, and interleukin-1β. Cyclin-dependent kinase inhibitor 1A (CDKN1A), a member of the Cip/Kip family, is positively correlated with the inhibition of genes related to cell cycle progression and the induction of senescence-associated genes (d'Adda di Fagagna, 2008; Kreis et al., 2019). Lamas-Paz et al. (2025) discovered through data analysis from various patient cohorts and mouse models that the expression level of CDKN1A is significantly associated with non-alcoholic steatohepatitis (NASH), liver fibrosis, and more severe liver diseases such as cirrhosis and hepatocellular carcinoma, with its overexpression exacerbating lipid metabolic disorders and inflammatory responses. Mice with a knockout of CDKN1A exhibit enhanced tolerance to metabolic liver injury, characterized by attenuated liver damage, reduced cell death, inhibited fibrotic progression, and improved lipid metabolism.

The member 12A of the tumor necrosis factor receptor family (TNFRSF12A) plays a critical role in cholestatic liver disease. Experiments have shown that hepatocyte apoptosis is closely associated with the expression of TNFRSF12A, and inhibiting TNFRSF12A expression can alleviate liver damage (Liao et al., 2023). Hepatocyte growth factor activator (HGFAC) is secreted by the liver, circulating in plasma and activated in injured tissues and tumors (Fukushima et al., 2018). The expression level of HGFAC is negatively correlated with the methylation of its promoter region, suggesting that HGFAC expression may be regulated by DNA methylation. Furthermore, the reduction in HGFAC expression is significantly associated with a shortened survival period in liver cancer patients (Yin et al., 2019).

### 4.2 Research progress on candidate compounds for NAFLD treatment

This study suggests that Cyclosporine may represent a highly promising novel candidate compound for the treatment of NAFLD. Cyclosporine, as a calcineurin inhibitor, primarily functions to suppress inflammatory responses and alleviate symptoms related to irritation (Zhong et al., 2020). Clinically, this drug is widely utilized to prevent graft-versus-host disease (GVHD) post-transplantation and to treat various autoimmune disorders (Xie et al., 2024). In recent years, studies have indicated that the potential therapeutic applications of Cyclosporine in liver diseases have garnered extensive attention. Studies have reported (Halestrap and Davidson, 1990) that Cyclosporine can inhibit significant Ca^2+^-induced mitochondrial swelling in the liver and heart, a mechanism likely associated with its regulatory effects on intracellular calcium homeostasis. Calcium ions play a pivotal role in cellular signal transduction and metabolic regulation, and their abnormal elevation is closely associated with the pathogenesis of various liver diseases. Kobayashi et al. (2022) reported that the immunosuppressive agent Cyclosporine A (CsA) confers notable neuroprotection in models of ischemic and traumatic brain injury. This observation supports CsA’s established role in immune modulation and implies its potential hepatoprotective capacity, possibly through the regulation of apoptotic and autophagic pathways. Additionally, there is evidence to suggest that CsA could inhibit the progression of NAFLD by reducing oxidative stress within hepatocytes. Despite its documented hepatotoxicity, CsA emerged as the most promising repositioning candidate because it simultaneously targets the two hub genes CDKN1A and CXCL2 and exhibited one of the lowest docking free energies in our screen, indicating a high likelihood of direct, multi-pathway activity against NAFLD. The drug-vacant status of HGFAC, an extracellular serine protease that zymogen-activates hepatocyte-growth factor, marks this node as an attractive *de novo* target (Sargsyan et al., 2023). Its solvent-exposed activation loop and trypsin-fold catalytic cleft provide a tractable pocket for either covalent inhibitors or antibody blockade, opening a route for first-in-class therapy. Moreover, decades of pharmacokinetic data and recent advances in liver-targeted nano-formulations provide realistic avenues to administer CsA at micro-doses or with tissue-specific delivery, thereby minimizing systemic exposure and liver injury while preserving therapeutic efficacy. Nonetheless, the known hepatotoxic potential of CsA necessitates careful dose management in future studies. Recent metabolomic studies indicate a clear dose-dependent hepatotoxic effect of CsA: oral micro-doses (<5 mg kg^-1^ day^-1^) administered for 4 weeks resulted in mild, reversible biochemical changes in rats, whereas higher doses (≥10 mg kg^−1^ day^−1^) significantly elevated circulating bile acids and induced hepatic lipid accumulation (Yen et al., 2024). Notably, hepatic lipid accumulation exacerbates NAFLD pathology, thus conflicting with CsA’s therapeutic objectives (Losurdo et al., 2018). Consequently, future NAFLD studies employing CsA should restrict dosing to sub-immunosuppressive micro-doses, accompanied by rigorous therapeutic drug monitoring and routine assessments of bile acids, ALT, and AST, thereby maximizing metabolic benefits while minimizing hepatotoxic risk.

### 4.3 Simultaneous treatment of liver and spleen, clearing heat and resolving dampness

The clinical manifestations of NAFLD primarily include nausea, abdominal distension, loss of appetite, and fatigue. These symptoms can be categorized in traditional Chinese medicine as “intercostal pain,” “phlegm syndrome,” and “accumulation.” The pathophysiological mechanisms of this disease are primarily characterized by liver dysfunction and impaired spleen transportation. This condition often exhibits a mixed pattern of deficiency and excess, accompanied by qi and blood deficiency, with the primary lesions located in the liver, while also involving the spleen and kidneys, internal damp-heat accumulation, and obstruction of the liver meridians. Therefore, the therapeutic principles should focus on promoting liver function, strengthening the spleen, and clearing heat while transforming dampness. At the molecular level, the chemokine CXCL2 drives neutrophil infiltration and local inflammatory “heat,” mirroring the TCM concept of damp-heat accumulation in the liver; CDKN1A-induced cell-cycle arrest leads to hepatocellular stagnation, corresponding to liver dysfunction; TNFRSF12A mediates bile-acid-triggered pyroptosis and cholestatic injury, echoing “dampness obstructing bile flow;” whereas HGFAC activates hepatocyte growth factor to facilitate hepatic regeneration, functionally akin to reinforcing the spleen to restore transformation and transportation.Collectively, these gene–symptom correspondences forge a mechanistic bridge between modern molecular pathology and traditional concepts, highlighting why formulas that clear damp-heat, soothe the liver, and fortify the spleen are rational strategies for correcting NAFLD-related gene dysregulation. The two core genes and five components identified in this study align with the therapeutic principles for this disease. Notably, source tracing analysis and molecular docking visualization indicate that Polygonum cuspidatum exhibits the highest correlation with this disease. Polygonum cuspidatum topped the herb–gene network and its signature stilbenes (resveratrol, polydatin) showed the strongest docking to NAFLD hub genes CDKN1A and CXCL2, while its classical function of “clearing damp-heat and invigorating blood” mirrors the disease’s inflammatory-metabolic block. Resveratrol suppresses SREBP-1c mRNA and upregulates PPAR-α, thereby diminishing lipid accumulation and hepatocellular pathology in fatty liver disease (Mo et al., 2018). Furthermore, Resveratrol effectively protects against high-fat-induced liver injury by inhibiting endoplasmic reticulum stress and promoting autophagy (Chen et al., 2020), thus offering new avenues for the treatment of non-alcoholic fatty liver disease. The component–gene associations obtained from the HERB database in this study are merely statistical inferences based on similarities between transcriptomic signatures; such inference cannot establish a definitive regulatory relationship between the five active TCM compounds and the hub genes. Future confirmation will require ligand-binding assays, CRISPR-based perturbations, and validation at the transcriptomic or proteomic level.

## 5 Conclusion

In summary, this study employed bioinformatics to investigate the differentially expressed genes and associated pathways in NAFLD. The findings suggest that Cyclosporine may serve as a novel compound for the treatment of NAFLD. Furthermore, TCMs such as Alpinia officinarum, Curcuma longa, Polygonum cuspidatum, Smilax glabra, Coptis chinensis, Stephania tetrandra, and Cornus officinalis may represent potential therapeutic agents. Nevertheless, experimental validation is still required, and we anticipate that ongoing advances in multi-omics technologies will accelerate translational progress in this field.

## Data Availability

The datasets presented in this study can be found in online repositories. The names of the repository/repositories and accession number(s) can be found in the article/Supplementary Material.
